# Hydraulic Strategy of Cactus Trichome for Absorption and Storage of Water under Arid Environment

**DOI:** 10.3389/fpls.2017.01777

**Published:** 2017-10-18

**Authors:** Kiwoong Kim, Hyejeong Kim, Sung Ho Park, Sang Joon Lee

**Affiliations:** Department of Mechanical Engineering, Pohang University of Science and Technology, Pohang, South Korea

**Keywords:** cactus, water absorption, water storage, water pathway, survival strategy

## Abstract

Being an essential component in various metabolic activities, water is important for the survival of plants and animals. Cacti grown in arid areas have developed intrinsic water management systems, such as water collection through spines, water absorption through trichome, and water storage using mucilage. The water collection method of cactus is well-documented, but its water absorption and storage strategies remain to be elucidated. Thus, this study analyzed the morphology and wettability of cactus trichomes by using advanced bio-imaging techniques and by performing *in vitro* experiments on an artificial system mimicking these structures, respectively. In addition, the *in situ* water absorption process through the trichome cluster was quantitatively visualized. This paper proposes a new bio-inspired technique for dew collection based on information about the water management strategies of cactus. This study discusses the underlying water absorption and storage strategies of cactus and provides the experimental database required to develop a biomimetic water management device.

## Introduction

Water is one of the most important elements not only for human beings but also for plants, because it is highly essential for various metabolic activities (Hanson and Hitz, 1982; Kim et al., 2016). In general land plants, only a small amount of water is used for growth and metabolism, and the most of the remaining water is lost by leaf transpiration (Waring and Running, 1978). Thus, plants living in arid areas have to manage water effectively for their survival. They need to absorb water effectively into their body and minimize loss of the absorbed water, besides minimizing water evaporation through transpiration. Fortunately, some plants, such as cacti, can live under such harsh conditions. In particular, cacti can collect water through their spines. They possess strategies for water collection from fog (Ju et al., 2012). Considering these features, several studies have introduced various cactus spine-inspired systems, such as micro-tip arrays for water collection (Ju et al., 2013, 2014; Cao et al., 2014; Heng et al., 2014), an oleophilic array for collection of micron-sized oil droplets (Li et al., 2013), and directional transportation of gas bubbles (Ma et al., 2015; Yu et al., 2016). The collected water is transported through the trichomes on the epidermis and then stored in the stem filled with mucilage. Cactus mucilage has high affinity to water and stores water exceeding its own weight (Nobel, 2003). However, the mechanism by which trichomes transport the collected water into the mucilage has not been completely understood.

**Figure 1:**
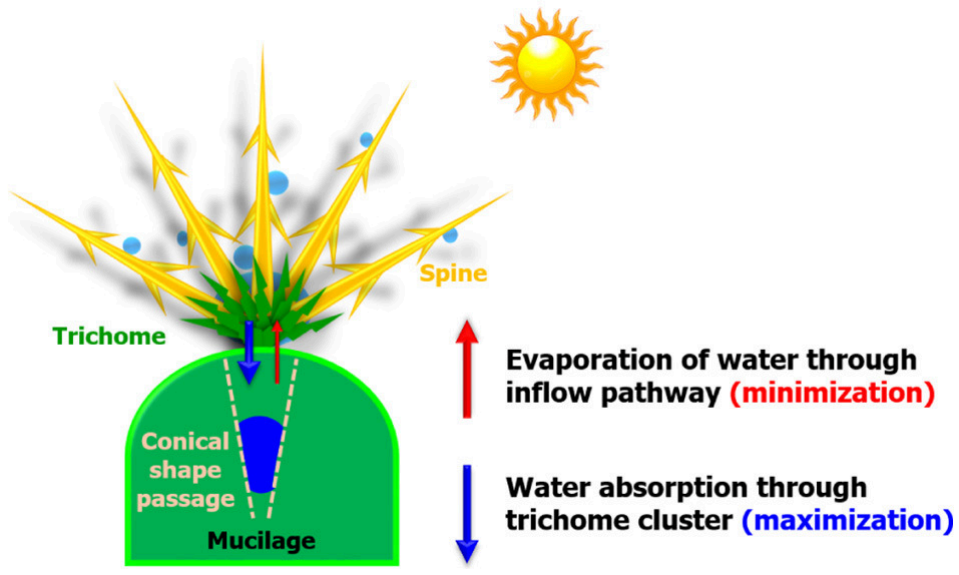
Schematic of survival strategies of cactus. When the sun rises, water droplets are condensed on spines and absorbed through the trichome cluster (downward direction; blue arrow). The absorbed water may be evaporated through the inflow pathway due to the strong heat and low water potential of the atmosphere. To survive in arid areas, cactus stem has to minimize the loss of water evaporation (upward direction; red arrow).

In the desert, large variations in temperature between day and night are observed. A fog usually forms at dawn and disappears soon after the sun heats up the air. Thus, cacti grown in the desert absorb fog moisture at dawn to survive (Ju et al., 2012). A large amount of collected water droplet is absorbed through the trichome cluster, and a small amount of absorbed water is evaporated through the inflow pathway even under high-temperature conditions (Figure 1). In other words, the condensed water droplets must be quickly absorbed before the sun rises, and the absorbed water have to be stored inside the stem. Many studies have focused on the water collection strategies of cactus spines, but their water absorption and storage processes remain poorly understood.

The present study experimentally investigated the effective water absorption and storage mechanisms of cactus. The morphological features of the trichome cluster were analyzed using advanced bio-imaging techniques. In addition, the rapid absorption process and water storage strategy of cactus were investigated through *in vivo* and *in vitro* experiments. The trichome surface contains hundreds of nanoscale grooves, and its roughness varies depending on the part. Contrary to our expectations, the trichome cluster has a hydrophobic surface with a contact angle of ~92°. The transition from Cassie state to Wenzel state occurs, when a large amount of water droplet is dropped on the trichome cluster. Surface roughness has been known to play an important role on the wettability of the surface, which can be divided into Cassie state and Wenzel state (Lafuma and Quéré, 2003). In the Cassie state, water droplet only sits on the peaks of the rough surface, and air pockets exist between the water droplet and rough surface. As the water droplet is evaporated, the transition from Cassie state to Wenzel state occurs. In the Wenzel state, the grooves or air space of the rough surface are wetted or filled with water (Manukyan et al., 2011). This feature maximizes water absorption and minimizes evaporation of the absorbed water through the inflow pathway. This type of one-way water transport is crucial for the design and fabrication of bioinspired water collection devices and for many other practical applications, including clothing and fluid control.

## Materials and methods

### Wettability test

The wettability of a cactus stem was examined using Nile red, a fluorescent hydrophobic probe. Nile red (Sigma–Aldrich, Korea) was prepared as a stock solution of 500 μg/mL in acetone. Its staining solution was made by adding 10 μL of the stock solution to 1 mL of 75% glycerol (Fowler and Greenspan, 1985; Greenspan et al., 1985). A sliced cactus stem was stained with the prepared Nile red solution for 30 min. The stained sample was illuminated by a light source with a wavelength in the range of 565–590 nm. Fluorescent images of the sample were observed by a fluorescence microscope (Zeiss Axiovert 200, Zeiss, Germany) attached with an optical long-pass filter (λ > 550 nm).

### Visualization of the conical-shaped passage

A cactus stem was sectioned transversely into several slices by using a microslicer (DTK-1000; Dosaka EM, Kyoto, Japan). The thickness of the sectioned slices was ~80 μm. The sliced samples were observed with an inverted microscope (Zeiss Axiovert 200, Zeiss, Germany) with a ×2.5 (NA = 0.075) objective lens. In addition, 2D sectional images along the depth direction were obtained using a two-photon laser scanning microscope (Leica Microsystems Ltd. TCS SP5 II MP with SMD, Germany) with a ×20 objective lens. The laser power was 1.9 kW (920 nm) and the total exposure time was 230 s. The field of view was 775 × 775 × 90 μm. The morphological structures of the sliced samples were consecutively captured at depth intervals of 1 μm. The acquired images were processed using the LAS AF 2.7 software (Leica Microsystems Ltd., Germany). Outlier noises were removed by using the Image J software (National Institutes of Health, USA) to improve image quality.

### Sample preparation and scanning electron microscope imaging

For SEM imaging, the sliced samples were freeze-dried at −84°C for 24 h using a freeze drying system (LABCONCO, USA). To avoid the charging effect on non-conducting surfaces, the freeze-dried samples were mounted on metal stubs and then coated with platinum (SC7640 model, Quorum Technology, UK) for 30 s. SEM images of the coated samples were captured by a SEM (JEOL JSM-7401 F, Japan) operating at the acceleration voltage of 15 kV.

### Fabrication of a cactus trichome-inspired system

A cactus trichome-inspired system was fabricated in this study. The system consists of double layers: the trichome-inspired nearly superhydrophobic membrane and the mucilage-inspired hydrogel layer. To fabricate the mucilage-inspired hydrogel, 0.2 wt% agarose gel was fabricated and squished. A fibrous paper (Kimberly-Clark Worldwide Inc., Korea) was used not only to cover the squished agarose gel but also to connect the two layers having opposite wettability characteristics. The trichome-inspired membrane was placed on the fibrous paper. As a trichome-inspired hydrophobic membrane, a polytetrafluoroethylene (PTFE) mesh (APEC Ltd., Korea) was selected for surface modification via plasma treatments, and a polyamide mesh (APEC Ltd., Korea) was used as a control model. By employing plasma treatment (Ryu et al., 2017) on the mesh, the surface of PTFE was modified to nearly superhydrophobic surface with a contact angle of ~149°. The surface morphology of the hydrophobic mesh was modified by plasma etching using Ar and O_2_ gases. A clean pristine hydrophobic mesh was loaded inside the vacuum chamber, and then the chamber was evacuated to the operating pressure of ~1.0 × 10^−1^ Torr. Surface morphology was modified by plasma excitation at a radio frequency (RF) of 13.56 MHz and a maximum RF power of 600 W. With varying regular periods of plasma treatment, the degrees of etching and wettability of the surface were changed (Figure S3). The structural features of the test samples were analyzed using a high-resolution FE-SEM (JEOL JSM-7401F, JEOL, Ltd., Japan).

### Analysis of the one-way transport artificial trichome system

To investigate the absorption property of the fabricated biomimetic system, a sessile deionized water droplet with 2 μL volume was dropped, and the shape variation of the droplet during evaporation process was temporally analyzed until the droplet was abruptly absorbed through the mesh (SmartDrop, Femtofab, Korea). The consecutively recorded images show the temporal evolution of water absorption through the mesh into the mucilage-inspired agarose gel. In addition, the evaporation rates through the three meshes were estimated from the temporal weigh variations of the mesh and mucilage-inspired agarose gel measured using a microbalance (Analytical Plus, AP250D, Ohaus Corp., Florham Park, NJ, USA). As a control group, the evaporation rate through a hydrophilic nylon membrane was measured.

## Results

### Structural characteristics of the trichome cluster of cactus

**Figure 2:**
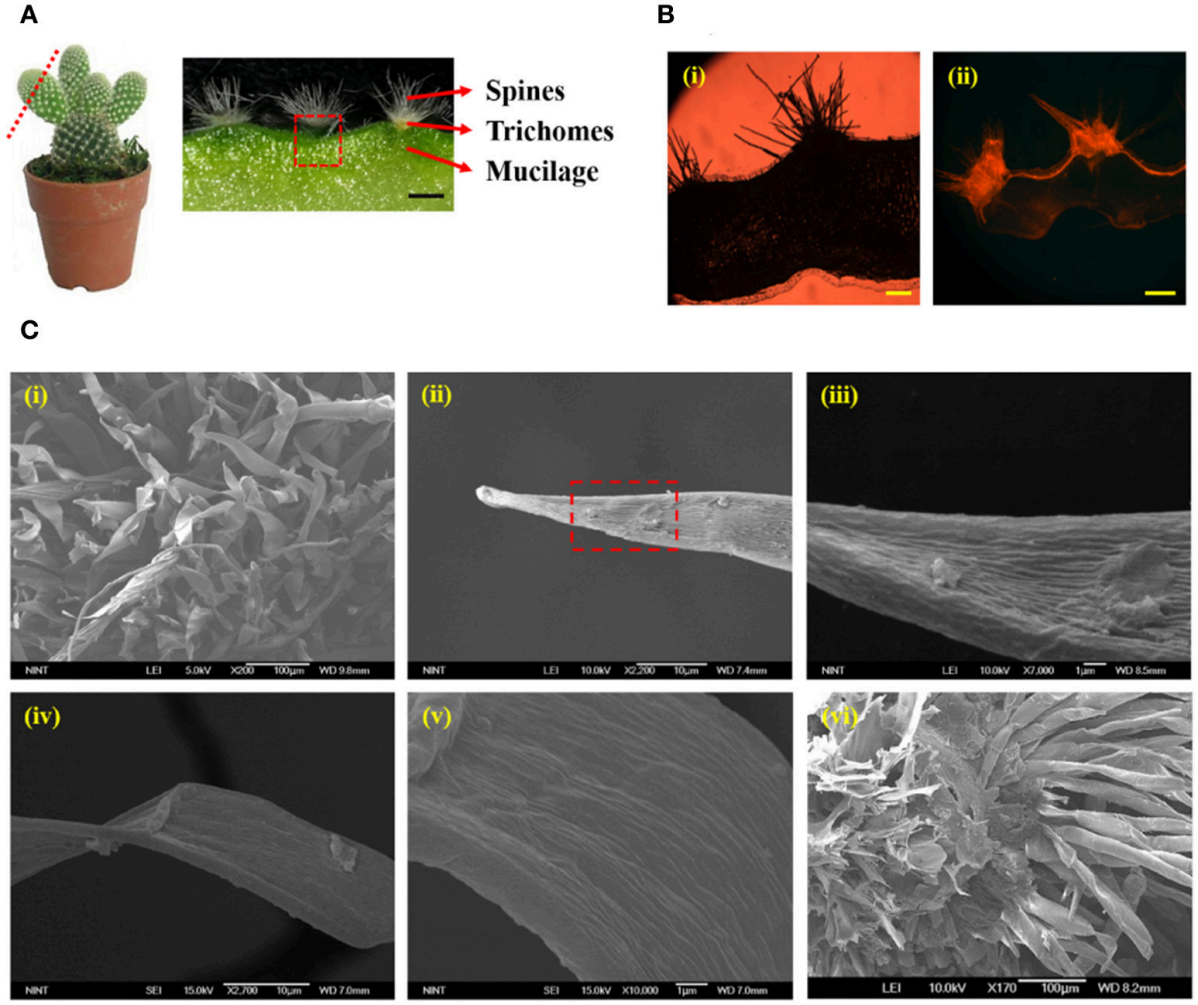
Morphological characteristics of cactus stem and trichomes. **(A)** Cross-sectional image of a cactus consisting of spines, trichomes, and mucilage. **(B)** Fluorescent images of the cactus stem **(i)** unstained and **(ii)** stained with Nile red, a hydrophobic fluorescence probe. The fluorescent parts, such as cactus spines, trichome cluster, and surface of the stem, have hydrophobicity. **(C) (i)** SEM image of spines and trichome cluster. **(ii)** The tip part of a trichome has a conical shape with an apex angle of 17°. **(iii)** Magnified image of the region marked by red dotted square in **(ii)**. Hundreds of nanogroves exist on the surface. **(iv)** The base part of the trichome also has **(v)** hundreds of nanogroves. However, its roughness is less than that of the tip part. **(vi)** Bottom view of the trichome cluster. All trichomes are adhered toward the center of the trichome cluster. Scale bars, 250 μm **(A)** and 200 μm **(B)**.

The structural characteristics of the cactus *Opuntia microdasys* (OM), which is a fog collector representative, were analyzed using various advanced imaging techniques. The stem of well-grown OM was sliced to 200 μm thickness. The optical image of the sliced OM stem is shown in Figure 2A. The cross-sectional image of a cactus stem exhibits three distinct parts: spines, trichomes, and mucilage with high affinity to water. To reveal the hydraulic survival strategy of the cactus, the hydrodynamic characteristics of trichomes were experimentally investigated. To check its wettability, the trichome cluster was stained with Nile red (Sigma–Aldrich, Korea), a fluorescent hydrophobic probe (Fowler and Greenspan, 1985; Lehnert et al., 2013). An unstained sliced cactus stem was observed to study its autofluorescence (Figure 2Bi). Any fluorescent part was not detected in the unstained cactus stem. This result indicates that the sliced cactus stem does not have autofluorescence at the 565–590 nm wavelength range. To facilitate the absorption of condensed water, the trichome cluster was supposed to be hydrophilic because a hydrophilic surface has a stronger adhesive force at the solid–liquid interface than a hydrophobic surface (Adamson and Gast, 1967). Unexpectedly, the fluorescence image of the trichome cluster stained with Nile red shows that the trichome cluster and stem surface of the cactus are hydrophobic. The hydrophilic mucilage (Ogburn and Edwards, 2009), which occupies most of the cactus stem, was not stained by Nile red solution. The trichome cluster was carefully attached on a slide glass, and then a saturated fog was applied to build up a droplet on the trichome surface. The shape of the water droplet was captured by a CCD camera (QIMAGING-Q42286, Canada) and the contact angle of water droplet on the trichome surface was measured using the Image J software (National Institutes of Health, USA). As a result, the contact angle of water droplet on the surface near the trichome tip is about 92° (Figure S1).

The detailed morphological structure of the trichome was observed under a scanning electron microscope (Figure 2C). The trichome has a conical shape with an apex angle of 17°, and spines are positioned in the gaps of the trichomes (Figure 2Ci). The length of each trichome ranges from 100 to 120 μm, and the diameter at the base part ranges from 30 to 40 μm. In addition, the tip part of the trichome has a rougher surface compared with the base (Figures 2Cii–v). Figure 2Cvi shows the bottom view of the trichome cluster. All trichomes are attached toward the center of the trichome cluster. These results support that condensed water droplets can be abosrbed into the cactus stem with the help of surface-free energy and capillary force gradients. Thus, integration of these features would help to absorb the condensed water droplets on the spines.

### Water droplets on the trichome cluster

**Figure 3:**
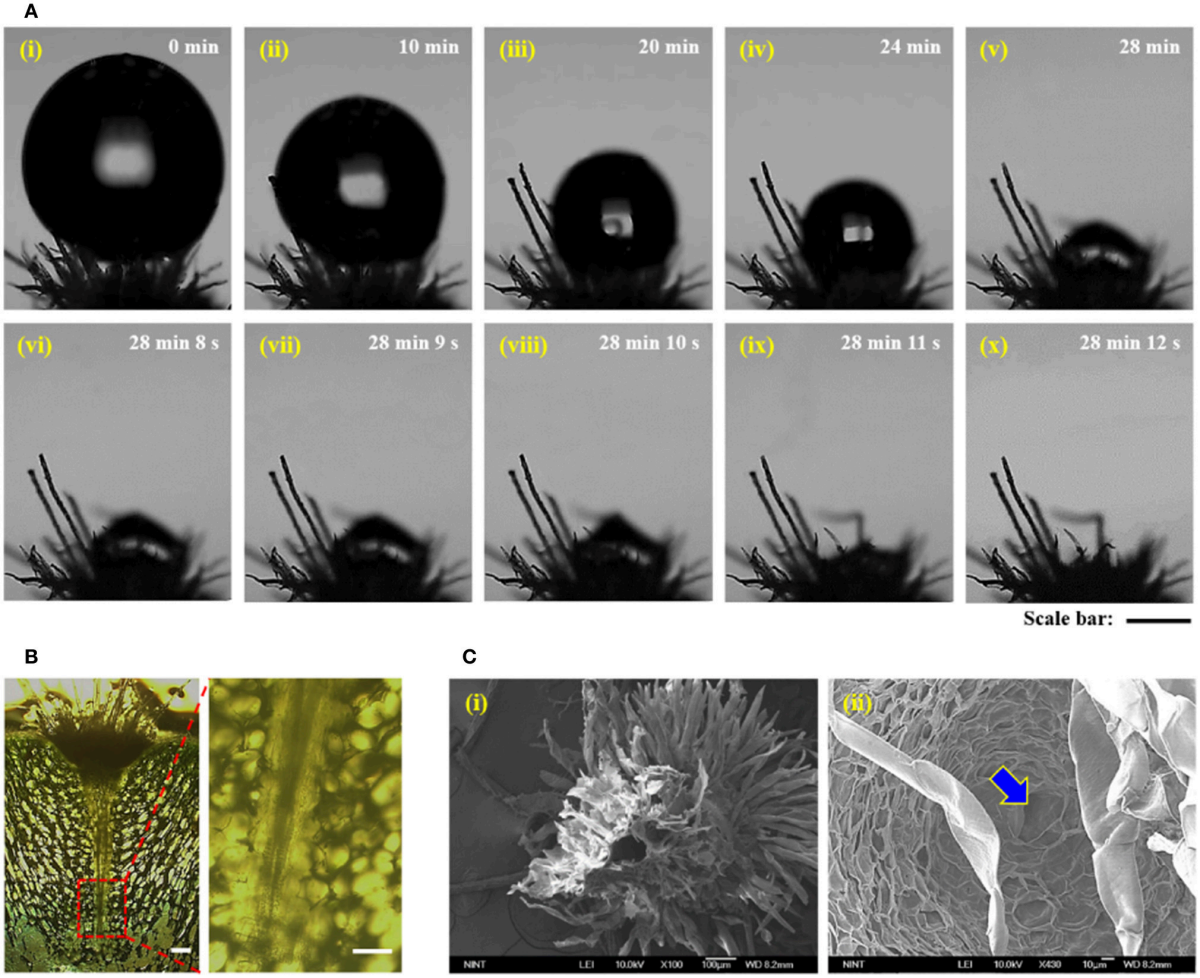
Hydrodynamic features of the trichome cluster and conical-shaped pathway. **(A)** *In situ* optical microscopic images showing evaporation and absorption of water droplet ~0.5 μL in volume dropped on the trichome cluster. **(i)** A water droplet is deposited on trichomes in Cassie state due to its wettability. **(ii–vii)** With continuous water evaporation, the contact line of the water droplet begins to move downward until the droplet transforms into Wenzel state. **(viii–x)** Finally, the water droplet is rapidly absorbed into the mucilage in a few seconds. **(B)** *In vivo* optical microscopic images of a sliced cactus stem. A conical-shaped pathway exists under the trichome cluster (left). Magnified image of its sharp end (right). **(C) (i)** SEM image of the conical-shaped pathway in lyophilized trichome cluster. **(ii)** The pathway starts from the trichome cluster marked by blue arrow. Scale bars, 300 μm **(A)** and 30 μm **(B)**.

A water droplet with ~0.5 μL volume was dropped on the trichome cluster (Figure 3A). Spines were removed to focus on the interaction between the trichome cluster and water droplet. Figure 3Ai shows the initial state of water droplet on trichomes. Air gap exists between the water droplet and the base part of the trichomes, indicating Cassie state (Lafuma and Quéré, 2003). With time, the droplet shrinks due to evaporation and slowly permeates into the air gap for tens of minutes (Figures 3Ai–vii). When the water droplet contacts the mucilage positioned just under the base part of the trichome cluster, immediate absorption occurs within a few seconds (Figures 3Aviii–x, Movie S1). This observation indicates transition from Cassie state to Wenzel state. In comparison with the time-consuming evaporation process (~28 min), the absorption process is very fast (~2 s). This finding implies that the combination of trichomes and mucilage of cactus can be considered as hydrophobic/hydrophilic double layers. Thus, fast absorption occurs when water droplets on a hydrophobic surface contact the hydrophilic suface just under the hydrophobic layer.

A conical-shaped pathway exists under the trichome cluster (Figure 3B). This pathway ranges from 130 to 150 μm with an apex angle of 7–10° (Movies S2, S3). The conical-shaped structure is connected to vascular bundles, and structures composed of two cones are occasionally discovered (Figure S2). Figure 3C shows the bottom view of the lyophilized trichome cluster. A hollow hole is shown in the center region. This feature indicates that this space would be filled with mucilage before the lyophilization (Figure 3Ci). Thus, the water can be possibly absorbed when condensed water droplets reach to the muciliage positioned just under the trichome cluster. A pore is observed just under the trichome cluster (Figure 3Cii). Therefore, the absorbed water can be transported through the conical-shaped pathway not only to reach vascular bundles but also to supply water to surrounding parts.

### *In vitro* water absorption experiment using a cactus-inspired model

**Figure 4:**
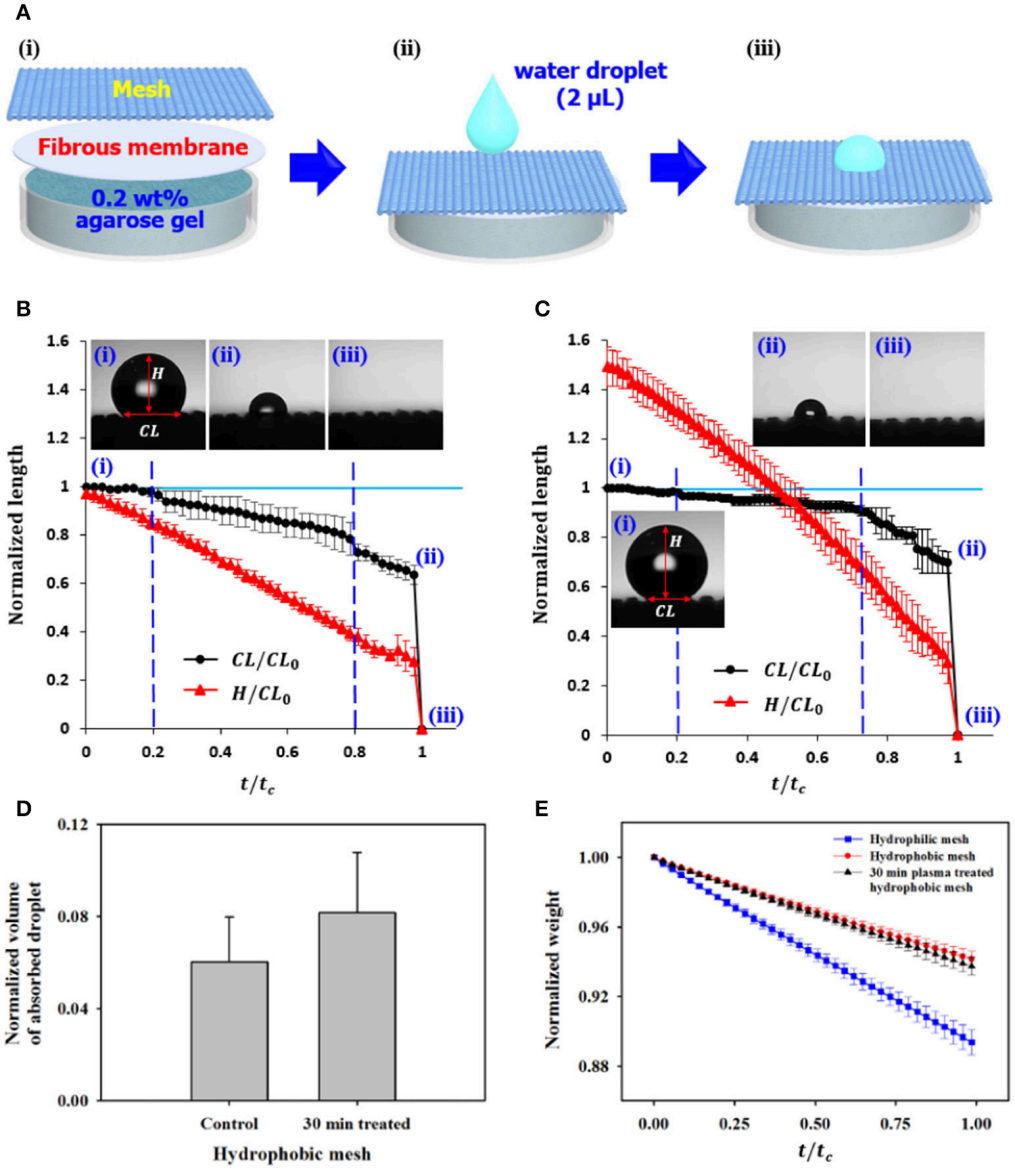
*In vitro* absorption and evaporation of water droplet on the surface of cactus-inspired model. **(A)** Schematic of the experimental setup. Three different types of meshes with different wettability were tested. A water droplet with 2 μL volume was dropped on the mesh. Temporal variations in the shape and absorption process of the water droplet were continously observed on the **(B)** hydrophobic mesh and **(C)** the plasma-treated hydrophobic mesh. **(i)** At the initial state, the droplet is dropped on the mesh. With time, the droplet is evaporated and permeated into the mesh. Images of the water droplet **(ii)** just before and **(iii)** after the water absorption. **(D)** Comparision of normalized volumes of absorbed water droplet on the different types of meshes. **(E)** Temporal variations of normalized weight of the Petri dish containing the three meshes. Error bars indicate standard deviation (*n* = 2).

To further understand water management strategies of cacti, a cactus-inspired double-layered water absorption *in vitro* model was designed. The hydrophobic trichome cluster was imitated by a porous mesh (APEC Ltd., Korea), and 0.2 wt% agarose gel (Sigma–Aldrich, Korea) was placed in a Petri dish to simulate the hydrophilic mucilage. In addition, a fibrous membrane (Kimberly-Clark Worldwide Inc., Korea) was inserted between the mesh and agarose gel for stable connection of the two layers having opposite wettability characteristics (Figure 4Ai). After dropping a water droplet with 2 μL volume on the mesh, its shape variation and absorption process were continously observed (Figures 4Aii,iii).

Three different types of meshes were tested: hydrophilic mesh (control; APEC Ltd., Korea), hydrophobic mesh, and 30 min plasma-etched hydrophobic mesh (Table 1). For the case of hydrophilic mesh (M1) with a contact angle of ~24°, the dropped water droplet is rapidly absorbed as soon as it contacts the mesh (Movie S4). However, when two types of hydrophobic meshes are used, the water droplet stays for a while on the surface and is absorbed after a period of time.

**Table 1:** Physical properties of the three meshes tested in this study.

	**Contact angle (°)**	**Pore size (μm)**	**Thickness (μm)**
Hydrophilic mesh (M1)	24	207	278
Hydrophobic mesh (M2)	131	210	230
30 min plasma-etched hydrophobic mesh (M3)	149	210	223

For quantitative analysis, the length of contact line (*CL*) and height (*H*) of water droplet were measured from recorded movies and normalized with the initial *CL* length (*CL*_0_). These parameters were consecutively measured as a funcion of *t/t*_*c*_, where *t* is the length of time after a water droplet drops on the mesh surface and *t*_*c*_ is the total time from the dropping to the completing droplet absorption (Figures 4B,C). Two transition points, marked by blue dashed lines, are found during water evaporation and absorption. Figure 4Bi shows the initial state of the water droplet when it was dropped on the hydrophobic mesh (M2). First, the water droplet starts to evaporate. The normalized *CL* length (*CL/CL*_0_) of water is nearly constant, which is defined as the constant *CL* (*CCL*) phase. During the *CCL* phase, the normalized *H* (*H*/*CL*_0_) is gradually decreased until the normalized time (*t/t*_*c*_) is about 0.2. At the second phase, the normalized *CL* length starts to decrease with an average decreasing rate of −0.31, and the normalized *H* is continously decreased at the same rate as the first phase. The transition from Cassie state to Wenzel state starts from the second phase. The decreasing rate of the normalized *CL* length becomes slightly steep (~−0.35) beyond the second transition point (*t/t*_*c*_ ~0.8), whereas that of the normalized *H* remains consistent. With continuous evaporation and downward direction movement, the dropped water droplet reaches the agarose gel at a certain point and then the water droplet is absorbed instataneously (Figures 4Bii,iii, and Movie S5).

For the 30 min plasma-etched hydrophobic mesh (M3), which has a higher contact angle than the hydrophobic mesh, the normalized *H* is around 1.5 at the initial state (Figure 4C). Similar to the hydrophobic mesh (M2), the three phases are clearly observed. The initial normalized time of *t/t*_*c*_ = 0.2 represents the *CCL* phase, whereas the normalized *H* is steadily decreased. However, the decreascing rate of the normalized *CL* after the first transition point is about −0.19, which is lower than that of the M2 mesh. In addition, the second transition point shifts forward from 0.8 to 0.73. After the second transition point, the normalized *CL* length is decreased with a high rate of −0.74, and then the water droplet is abruptly absorbed (Figures 4Cii,iii, and Movie S6). As a result, the normalized volume of the absorbed droplet on the 30 min plasma-treated hydrophobic mesh M3 is higher (~0.08) than that of the hydrophobic mesh M2 (~0.06) (Figure 4D).

To compare the evaporation rates for the three meshes, the weight variations of the Petri dish containing mesh, fibrous membrane, and agarose gel were continously monitored for 1 h as a funtion of *t/t*_*c*_, where *t* is the duration of the experiment and *t*_*c*_ is the total time (Figure 4Ai). The decreasing rate of the normalized weight on the hydrophobic mesh (M2) is about −0.06, and that of the 30 min plasma-treated hydrophobic mesh (M3) is about −0.07 (Figure 4E). However, the decreasing rate of the normalized weight (~−0.11) on the the hydrophilic mesh (M1) is higher than that on the hydrophobic case mesh.

## Discussion

The trichome cluster of a cactus stem has unique structural features to survive in harsh arid areas. As shown in Figure 2C, spines are located in the gaps of the trichome cluster. In addition, the spines and trichomes are attached toward the center of the trichome cluster. As a result, capillary force can be utilized to move the condensed water droplet into the cactus stem, because trichomes are non-parallel to each other. In addition, the Laplace pressure gradient is caused by the trichome's cone shape and the surface energy gradient. It generates a certain driving force (Lorenceau and Quéré, 2004). This result is similar to the function of cactus spines for the directional movement of water droplets toward the epidermis of cactus stem (Ju et al., 2012).

In general, hydrophilic surfaces have high affinity to water (Adamson and Gast, 1967). Thus, the hydrophilic surface is advantageous in obtaining moisture from the atmosphere. However, the collected water droplets have to be rapidly absorbed into the stem, because the surrounding temperature is high and humidity is low in arid areas and deserts, which favors rapid evaporation. The directional water transport is induced by the hydrophobic/hydrophilic double layers (Wu et al., 2012), because heterogeneous surface tension can induce a driving force to assist the directional transport of water (Wang et al., 2010).

To achieve fast water absorption, water evaporation procedure is not always required. When a water droplet forms a certain shape which can induce Wenzel state on the trichome surface in any way, it can be abruptly absorbed into the stem. As discussed in Figure 2B, the trichome cluster has a hydrophobic surface. Thus, the cactus stem can be considered to have double layers consisting of hydrophobic(trichomes)/hydrophilic(mucilage) materials. Water droplets can penetrate spontaneously from the hydrophobic layer toward the hydrophilic layer. Conversely, the opposite directional transport is strictly restricted because the absorbed water is blocked and spread into the hydrophilic region (Tian et al., 2014). In a previous study, the reason for this phenomenon was theoretically analyzed. The anisotropic critical breakthrough pressure (Pc) is larger in the downward direction (from hydrophobic to hydrophilic) compared with the upward direction (from hydrophilic to hydrophobic) because of the coupling effect (Tian et al., 2012). If the wettability of the trichome cluster is hydrophilic, then the trichome cluster is better for collecting water from air. However, it is difficult to minimize evaporation of absorbed water due to its relatively high evaporation rate (Figure 4E).

The proposed nature-inspired *in vitro* system is easy to fabricate and can be utilized in the design of efficient water absorption and storage system. In addition, the present results are useful not only for understanding the underlying water absorption and storage strategies of cacti but also for providing the experimental data required to develop a new biomimetic water collection device.

## Author contributions

KK and SL proposed the study. KK, HK, and SP developed and performed the experiment. KK and HK analyzed experimental data and processed images. All authors discussed the results. KK wrote the paper. All authors participated in completing the manuscript.

### Conflict of interest statement

The authors declare that the research was conducted in the absence of any commercial or financial relationships that could be construed as a potential conflict of interest.
