# Supplementary material for: Hydraulic Strategy of Cactus Trichome for Absorption and Storage of Water under Arid Environment
Source: Front Plant Sci. 2017 Oct 18;8:1777. doi: 10.3389/fpls.2017.01777 (PMC5651663; doi:10.3389/fpls.2017.01777)
Supplement: Supplementary file 7 [file DataSheet1.docx]

**Hydraulic strategy of cactus trichome for absorption and storage of water under arid environment**

Kiwoong Kim^1^, Hyejeong Kim^1^, Sung Ho Park^1^, and Sang Joon Lee^*, 1^

^1^Department of Mechanical Engineering, Pohang University of Science and Technology (POSTECH), San 31, Hyoja-dong, Pohang 790-784, Republic of Korea

^*^Corresponding author

Tel: +82-54-279-2169, Fax: +82-54-279- 3199, E-mail: [sjlee@postech.ac.kr](mailto:sjlee@postech.ac.kr)

**Supplementary information**


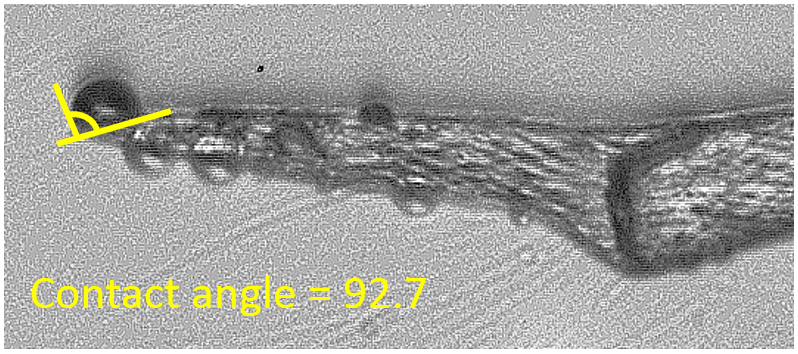


**Figure S1. Contact angle at the tip of a cactus trichome.**


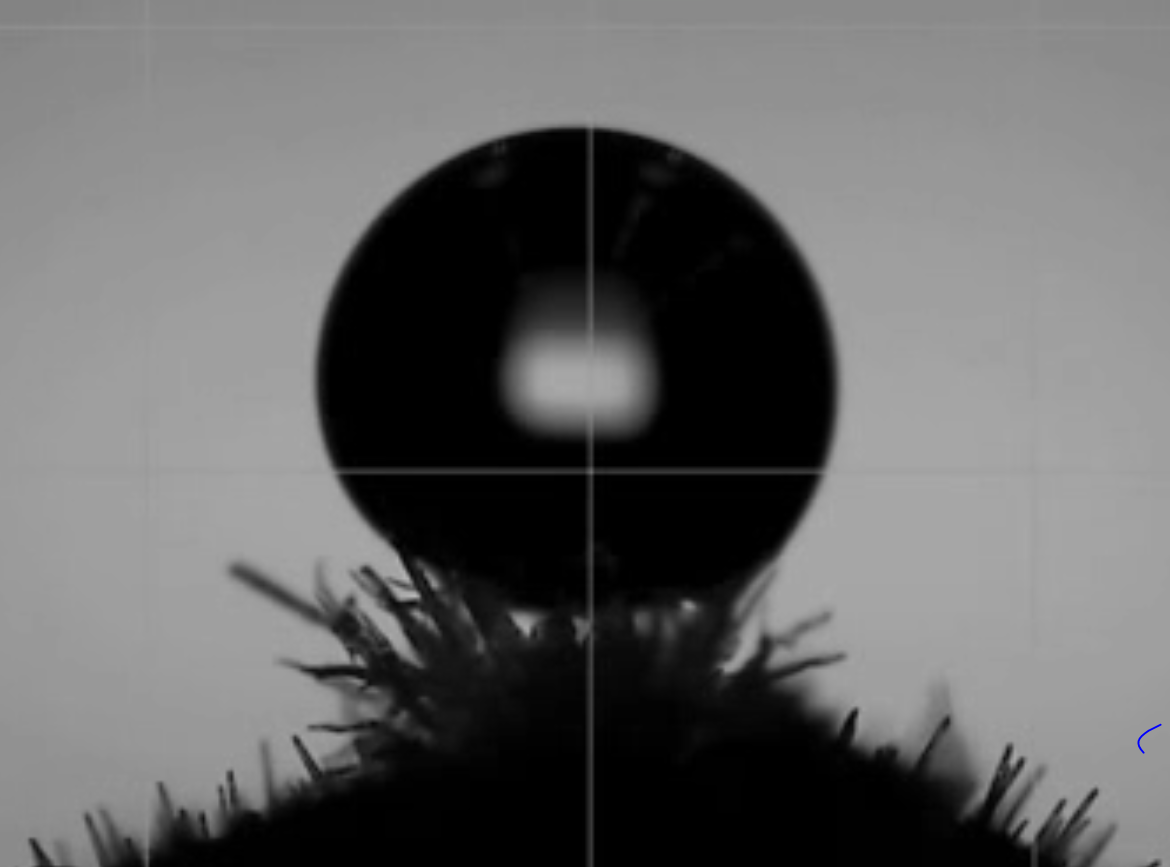


**Movie S1. Evaporation and absorption of a water droplet on the spine-removed trichome cluster.**


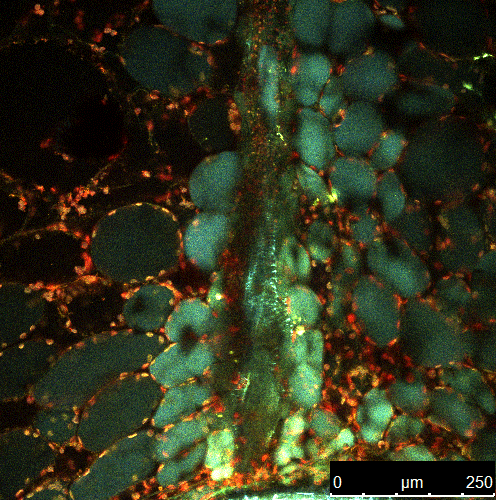


**Movie S2. Internal morphological image structure of cactus stem with a conical-shaped structure.**


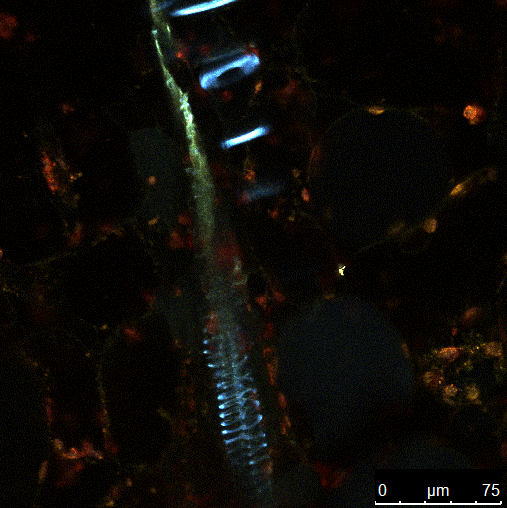


**Movie S3. Tip part of the conical-shaped structure.**


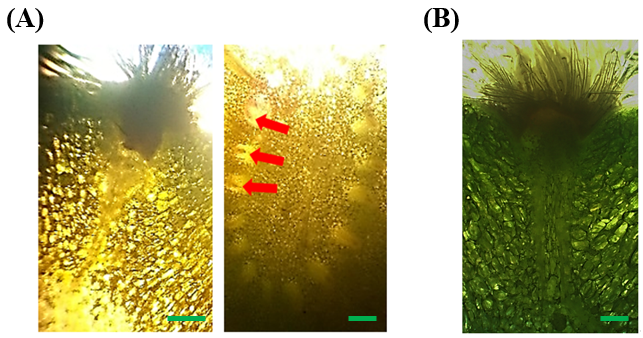


**Figure S2. (A) The conical-shaped structure is directly connected to vascular bundle. (B) Two conical shapes under the trichome cluster. Scale bars, 120 μm (A) and 30 μm.**


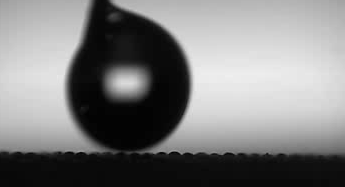


**Movie S4. Water droplet is absorbed at the instant when it contacts the hydrophilic surface (M1).**


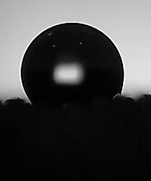


**Movie S5. Water droplet absorption on the hydrophobic mesh (M2).**


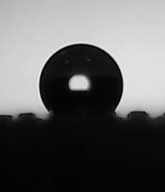


**Movie S6. Water droplet absorption on the 30 min plasma-treated hydrophobic mesh (M3), which has a higher contact angle than the hydrophobic mesh (M2).**


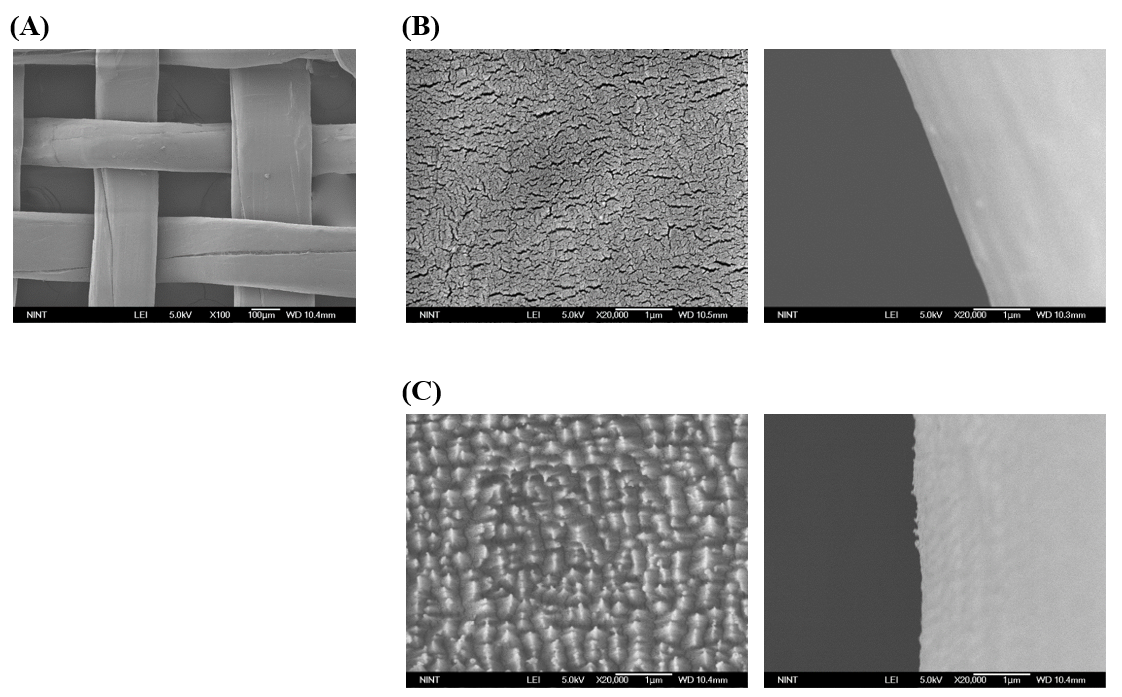


**Figure S3. (A) Top view of the hydrophobic mesh (60 mesh). (B) Magnified surface image of the hydrophobic mesh (M2) with a boundary line. (c) Magnified surface image of the 30 min plasma-treated hydrophobic mesh (M3) with a boundary line.**
